# Distillation of crop models to learn plant physiology theories using machine learning

**DOI:** 10.1371/journal.pone.0217075

**Published:** 2019-05-29

**Authors:** Kyosuke Yamamoto

**Affiliations:** SoftBank Corp., Tokyo, Japan; Pennsylvania State University, UNITED STATES

## Abstract

Convolutional neural networks (CNNs) can not only classify images but can also generate key features, e.g., the Google neural network that learned to identify cats by simply watching YouTube videos, for the classification. In this paper, crop models are distilled by CNN to evaluate the ability of deep learning to identify the plant physiology knowledge behind such crop models simply by learning. Due to difficulty in collecting big data on crop growth, a crop model was used to generate datasets. The generated datasets were fed into CNN for distillation of the crop model. The models trained by CNN were evaluated by the visualization of saliency maps. In this study, three saliency maps were calculated using all datasets (case 1) and using datasets with spikelet sterility due to either high temperature at anthesis (case 2) or cool summer damage (case 3). The results of case 1 indicated that CNN determined the developmental index of paddy rice, which was implemented in the crop model, simply by learning. Moreover, CNN identified the important individual environmental factors affecting the grain yield. Although CNN had no prior knowledge of spikelet sterility, cases 2 and 3 indicated that CNN realized about paddy rice becoming sensitive to daily mean and maximum temperatures during specific periods. Such deep learning approaches can be used to accelerate the understanding of crop models and make the models more portable. Moreover, the results indicated that CNN can be used to develop new plant physiology theories simply by learning.

## Introduction

Recently, machine learning has experienced tremendous advancements. Deep learning has provided solutions to many tasks that could not be solved by conventional machine learning. One remarkable achievement of deep learning is AlphaGo [1] (developed by DeepMind), a computer program that plays the game Go and can beat professional human Go players without any handicaps.

Convolutional neural networks (CNNs), which are typically used in image processing tasks and AlphaGo, represent a powerful deep learning method. The remarkable accuracy of CNNs was demonstrated when they outperformed conventional image processing in ImageNet Large Scale Visual Recognition Challenge 2012 (ILSVRC-2012) [2–5]. In addition to their accuracy, an interesting feature of CNNs is that they do not require feature engineering. Instead, CNNs alone generate the key features used for classifying images from the input, and then classify the images using these features. For instance, a Google CNN learned to detect cats after being trained by watching YouTube videos.

Machine learning, including deep learning, can also be used for agricultural purposes. Most researches utilizing machine learning in agriculture have focused on image processing tasks, e.g., weed detection and identification [6, 7], disease detection [8–14], pest identification [15–17], stress phenotyping [18], internode length estimation [19], vegetation area detection [20], flower detection [21], leaf counting [22], and fruit detection [23–27]. However, machine learning can be utilized as an alternative approach for discovering embedded knowledge that may be present in a dataset [28]. This type of approach includes maize yield prediction using decision tree models [29]; comparison of machine learning methods for the yield prediction of peppers, beans, corns, potatoes, and tomatoes [30]; support vector machine (SVM)-based crop models for paddy rice [31]; identifying important environmental features for the maize production using random forests [32]; evaluation of relations between the meteorological factors and the rice yield variability using conditional inference forests [28]; identification of important variables for modeling Andean blackberry production using artificial neural networks [33]; and predicting the photosynthetic capacity of leaves using partial least squares regression [34].

As described above, machine learning has already been used to predict crop growth from environmental information. However, there are several problems associated with such research designs. First, data collection in agriculture is a challenging task. Machine learning, especially deep learning, requires a huge amount of data to produce a model with high generalizing capability [14]. For instance, in ILSVRC-2012, 1.2 million images were provided for training. Given the various growing times of important crops, it may take a year to collect a single dataset. For this reason, research that attempts to utilize machine learning for crop growth prediction tends also lacks sufficient data. One way to solve this problem is to use crop models [35–39] that can generate a huge number of datasets on crop growth via simulation.

Crop models and machine learning models differ greatly in how the models are constructed. Crop models perform an abstraction of the dynamic mechanisms of a plant’s physiological stages by fitting them into a mathematical model [30, 40], whereas machine learning produces models based on statistical theories and does not require any prior knowledge about physical mechanisms [30]. To date, there has been no study discussing the relations between machine learning models and crop models. If such a relation could be found, it would suggest that meaningful plant physiology models can be generated by machine learning. It would also mean that machine learning can develop new plant physiology theories by learning, which is similar to the Google AI that learned to identify cats. In this context, a previous study [14] showed that CNNs can use the visual cues employed by an expert rater to identify and quantify the symptoms of plant diseases.

In this research, a crop model has been distilled by applying a CNN to big data from crop growth generated by the crop model. The generated model is then analyzed by deep learning to find relations between the crop model and the deep learning model. Finally, the ability of deep learning to find the plant physiology knowledge behind the distilled crop model from the given data is discussed.

## Materials and methods

### Crop model

Paddy rice is a key crop in Asian countries, and thus, many crop models for paddy rice have been developed to date [35, 41–45]. SImulation Model for RIce-Weather relations (SIMRIW) [35] is a simplified process model for simulating the growth and yield of irrigated rice in relation to weather. In comparison with other crop models, SIMRIW requires less parameters to be provided in advance; hence, it is applicable to a wide range of environments [35]. Furthermore, SIMRIW requires adjusted parameters for specific rice varieties, but these have already been studied for the major rice varieties in Japan [46].

SIMRIW is a simplified process model for simulating the potential growth and yield of irrigated rice in relation to temperature, solar radiation, and CO_2_ concentration in the atmosphere [35]. The model is based on the principle that the grain yield *Y*_*G*_ of a crop forms a specific proportion of the total dry matter production *W*_*t*_:

$$\begin{array}{c} Y_{G}=hW_{t}, \end{array}$$ (1)

where *h* is the harvest index.

In SIMRIW, *W*_*t*_ is determined by the amount of short-wave radiation absorbed by the canopy. This relation is described as follows:

$$\begin{array}{c} \Delta W_{t}=C_{s}S_{s} \end{array}$$ (2)

where *C*_*s*_ is the conversion efficiency of absorbed short-wave radiation and *S*_*s*_ is the daily total absorbed radiation.

The developmental processes of rice crops are strongly influenced by environment and crop genotype [35]. In SIMRIW, these are described by the developmental index (*DV I*), which is defined as 0.0 at crop emergence, 1.0 at heading, and 2.0 at maturity.

*DV I* of day *t* is calculated by accumulating the developmental rate (*DV R*) until the day

$$\begin{array}{c} {DV\;I}_{t}=\sum_{i=0}^{t}DV\;R_{i} \end{array}$$ (3)

Day length and temperature are the major environmental factors determining *DV R* [35]; hence, *DV R* at 0.0 ≤ *DV I* ≤ 1.0 is defined as

$$DV\;R=\left\{ \begin{array}{ccc} \frac{1}{G_{v}\left\{1+\exp\left[-A\left(T_{mean}-T_{h}\right)\right]\right\}} & DV\;I\leq DV\;I^{*} & \left(4\right)\\ \frac{1-\exp[B\left(L-L_{c}\right)]}{G_{v}\left\{1+\exp\left[-A\left(T_{mean}-T_{h}\right)\right]\right\}} & DV\;I>DV\;I^{*},L\leq L_{c} & \left(5\right)\\ 0 & DV\;I>DV\;I^{*},L>L_{c} & \left(6\right) \end{array}\right.$$

where *T*_*mean*_ and *L* are daily mean temperature and day length, respectively. DV I* is the value of *DV I* at which the crop becomes sensitive to photoperiod, *L*_*c*_ is the critical day length, *T*_*h*_ is the temperature at which *DV R* is half the maximum rate at the optimal temperature, and *G*_*v*_ is the minimum number of days required for heading of a cultivar under optimal conditions. A and B are empirical constants.

*DV R* from heading to maturity (1.0 < *DV I* ≤ 2.0) is defined as

$$\begin{array}{c} DV\;R=\left\{1-\text{exp}\left[-K_{r}\left(T_{mean}-T_{\text{cr}}\right)\right]\right\}/G_{r}, \end{array}$$ (7)

where *G*_*r*_ is the minimum number of days for the grain-filling period under optimal conditions. K_r_ and T_cr_ are empirical constants.

The amount of absorbed radiation (*S*_*s*_) is a function of leaf area index (LAI). Daily dry matter production Δ*W*_*t*_ is calculated by multiplying the *S*_*s*_ value by an appropriate value of the radiation conversion efficiency *C*_*s*_ (Eq 2). *C*_*s*_ is constant for the fronthalf of the grain-filling stage, and decreases gradually toward zero for the back half:

$$\begin{array}{c} C_{s}=C_{0}*\frac{1+R_{m}\left(P-330\right)}{\left(P-330\right)+K_{c}} \end{array}$$ (8)

$$C_{0}=\left\{ \begin{array}{ccc} C & 0.0\leq DV\;I<1.0 & \left(9\right)\\ \frac{C(1+B)}{1+B\exp(\frac{DV\;I-1}{t})} & 1.0\leq DV\;I\leq 2.0 & \left(10\right) \end{array}\right.$$

in which *P* is CO_2_ concentration (ppm), *C*_0_ is the radiation conversion efficiency at 330 ppm CO_2_, *R*_*m*_ is the asymptotic limit of relative response to CO_2_, and K_c_, C, B, and t are empirical constants.

In SIMRIW, the harvest index *h* is defined as

$$\begin{array}{c} h=h_{m}\left(1-\gamma \right)\left\{1-\exp\left[-K_{h}\left(DV\;I-1.22\right)\right]\right\}, \end{array}$$ (11)

where *h*_*m*_ is the maximum harvest index of a cultivar under optimal conditions, K_h_ is an empirical constant, and *γ* is the percentage of spikelet sterility.

The harvest index decreases when the fraction of sterile spikelets increases or when crop growth stops before completing development due to cool summer temperatures or frost [35]. In SIMRIW, the effect of cool summer damage occurs in the period of the highest sensitivity of the rice panicle by cool temperatures (0.75 < *DV I* < 1.2) and can be described as follows:

$$\begin{array}{ccc} \gamma_{L} & = & \gamma_{0}-K_{q}Q_{t}^{a} \end{array}$$ (12)

$$\begin{array}{ccc} Q_{t} & = & \sum (22-T_{mean}), \end{array}$$ (13)

where *γ*_*L*_ is the percentage of sterility due to cool summer damage. *γ*_0_, K_q_, and a are empirical constants.

Sterile spikelets are also increased by high temperature at anthesis. In SIMRIW, this is described as follows:

$$\begin{array}{ccc} 1-\gamma_{H} & = & 1-1/\{1+\exp\left[-0.853\left(T_{H}-36.6\right)\right]\} \end{array}$$ (14)

where *γ*_*H*_ is the percentage of sterility due to high temperature at anthesis and *T*_*H*_ is the average daily maximum temperature (*T*_*max*_) at 0.96 < *DV I* ≤ 1.22. The actual spikelet sterility *γ* is calculated as the maximum of *γ*_*L*_ and *γ*_*H*_.

A schematic representation of the processes of growth, development, and yield formation of rice implemented in SIMRIW is shown in S1 Fig. Refer to S1 Table for details of the variables.

### Meteorological data acquisition

Meteorological data were obtained from the Agro-Meteorological Grid Square Data (hereinafter referred to as Grid Data) provided by the National Agriculture and Food Research Organization [47]. Grid Data provides daily data on air temperature, humidity, precipitation, and solar irradiance all over Japan with a 1-km resolution. The available data include past data from 1980 until present as well as forecast data for 26 days ahead.

Meteorological data from 1980 to 2016 on daily mean temperature, daily maximum temperature, and daily total global solar radiation were obtained from Grid Data. Day length was calculated based on the day of the year and latitude. Since CO_2_ concentration was not available in Grid Data, it was set as a constant value (350 ppm). Since environmental conditions were considerably similar within the 1-km interval, meteorological data were obtained for every 10 km. The meteorological data were divided by year and grid. Consequently, 132,460 datasets were obtained.

### Data generation using crop model

The meteorological data obtained from Grid Data were fed into SIMRIW to generate crop growth data. The parameters for Koshihikari, the most common paddy rice variety in Japan, were used for the SIMRIW simulation.

In most rice production areas in Japan, rice is planted in the beginning of May and harvested in the beginning of October of the same year. Therefore, the planting date was set as May 01 of the same year as meteorological data of each dataset. As a result, the amount of plant growth data obtained from SIMRIW was the same as the amount of meteorological data (132,460 datasets).

Climatic conditions are extremely different in the north and south parts of Japan. Therefore, in a few of the grids, In some of the grids, therefore, *DV I* increase was considerably slow in certain years or certain regions due to overly hot or cool temperatures. Datasets that did not reach 2.0 of *DV I* by October 05 of the same year as the planting date were excluded from further analysis.

### Distillation of crop model using CNN

The neural network design considered for this research is shown in S2 Fig. The first layer of the network was a 3 × 3 pixel convolutional layer with a stride of 1 × 1 pixel (in the horizontal and vertical directions) and padding of 2 × 2 pixel (in the horizontal and vertical directions). This convolutional layer mapped the single channel in the input to 32 feature maps using a 3 × 3 pixel kernel function. The second layer was a 3 × 3 pixel convolutional layer with a stride of 1 × 1 pixel and padding of 2 × 2 pixel, which mapped the 32 feature maps of the first layer to 64 feature maps. Rectified linear unit (ReLU) layers were adopted in all convolutional connected layers. The third and final layers were fully connected to produce a single value of prediction. Herein, the mean squared error (MSE) was used as a loss function. An Adam optimizer [48] was used to minimize error.

Meteorological data were shaped to a 2D array and fed into the CNN to obtain the prediction of the grain yield. The rows of the 2D array were related to days from the planting date, and the columns were related to the meteorological factors *L*, *T*_*mean*_, *T*_*max*_, *S*_*s*_, and *P*. Since the CNN architecture considered herein requires the same shape of inputs, zero padding in the vertical direction was applied when *DV I* reached 2.0 within 184 days from the planting date. Each meteorological factor was normalized before being fed into the CNN. The data were randomly split into 75% for training and 25% for validation. As shown in S3 Fig, the validation data were within the range of the training data, timewise and spatially. After the normalization, random noise ranging between -0.001 and 0.001 was added to the training and validation data (see S4 Fig and S2 Table for the results with different ranges of the random noise). The training was stopped when validation loss did not improve for 10 consecutive epochs.

Since the objective of this study was not the evaluation of accuracy, the model that produced the lowest validation loss was used for further analysis.

### Evaluation of the CNN model

There are several ways to evaluate and explain trained CNN models, e.g., layer visualizations and attention maps. In this study, saliency maps [49], a method concerning attention maps, were used to visualize the salient meteorological factors and timings that most contributed to grain yields.

Positive saliency, which increases the output (in this case, grain yield), was calculated based on the final dense layer of the CNN. Inputs used as initial seeds for the calculation were randomly selected from the training datasets (*n* = 500).

Three types of saliency were calculated in this study. First, saliency was calculated using all datasets. Next, datasets with higher *γ*_*H*_ or *γ*_*L*_ were extracted and used for saliency calculation to evaluate the differences in saliency when spikelet sterility occurred.

### Implementation

All calculations were made using Python 3.6 on an Ubuntu 16.04 Linux system. All experiments were executed on the Amazon Elastic Compute Cloud (EC2) with a single GPU of NVIDIA Tesla K80. SIMRIW is available as an R script [50]. In this study, the R script was ported to Python and used for data generation. The CNN model was implemented in Keras 2.1.5 [51]. The calculation of saliency maps was made using the keras-vis package [52].

Source codes developed for this research are available online (https://github.com/ky0on/simriw and https://github.com/ky0on/pysimriw). All the data collected and generated in this study are also available online [53].

## Results

### Crop modeling

**Fig 1:**
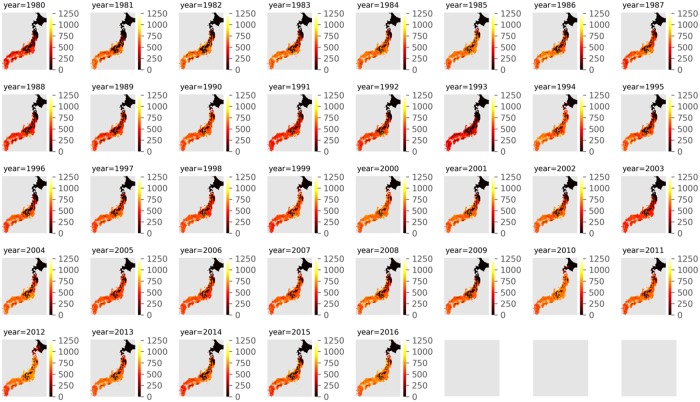
Heatmap of the grain yield simulated by SIMRIW. In northern areas of Japan, *DV I* does not reach 2.0 because of cold temperatures; hence, the grain yield is considerably low.

**Fig 2:**
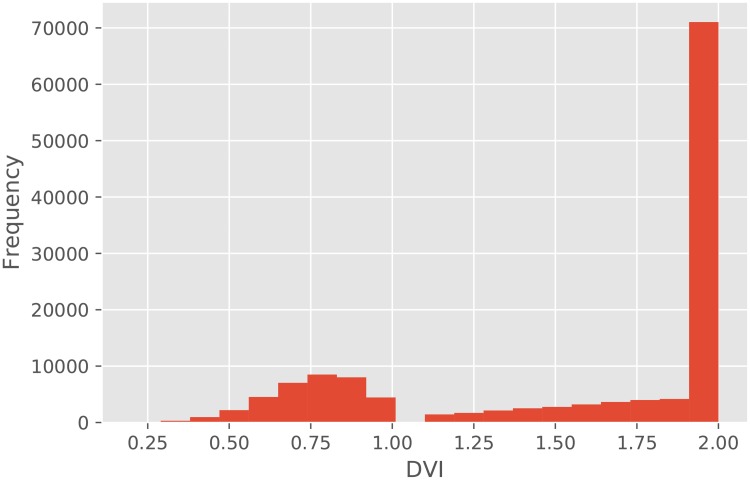
Histogram of final *DV I* of all datasets. In total, 48% of datasets do not reach 2.0 of *DV I*.

Fig 1 shows a heatmap of the grain yield simulated by SIMRIW. In northern areas of Japan, *DV I* does not reach 2.0 because of cold temperatures; hence, the grain yield is considerably low. Fig 2 shows a histogram of final *DV I* in all datasets. In total, 48% of datasets did not reach 2.0 of *DV I*.

Table 1 presents a summary of the harvest index considering *γ*_*H*_ ($h_{\gamma_{H}}$) and *γ*_*L*_ ($h_{\gamma_{L}}$). Datasets for which *DV I* does not reach 2.0 were eliminated in Table 1. The minimum values of $h_{\gamma_{H}}$ and $h_{\gamma_{L}}$ are 0.15 and 0.13, respectively. The 10th percentiles are 0.36 and 0.35, respectively. Datasets below the 10th percentile were used for saliency calculation to evaluate the effect of spikelet sterility due to high temperature at anthesis or cool summer damage.

**Table 1:** Summary of the harvest index considering *γ*_*H*_ and *γ*_*L*_, where final *DV I* ≥ 2.0.

	Mean	Std	Min	10%	25%	50%	75%	max
$h_{\gamma_{H}}$	0.37	0.01	0.15	0.36	0.37	0.38	0.38	0.38
$h_{\gamma_{L}}$	0.36	0.01	0.13	0.35	0.36	0.36	0.36	0.36

### Crop model distillation

CNN training stopped at epoch 55 and required 1 h for its completion. In the validation process, 3.1 ms ± 37 *μ*s were required to produce 10 predictions.

Loss during CNN training and validation are shown in S4 Fig. The loss is considerably improved in the first 20 epochs and slightly improved after 20 epochs in the training process. The loss in the validation process also improves as the epochs increase with fluctuations. Overall, the smallest validation loss is observed in the 45th epoch. The trained model of this epoch was thus used for further evaluation.

**Fig 3:**
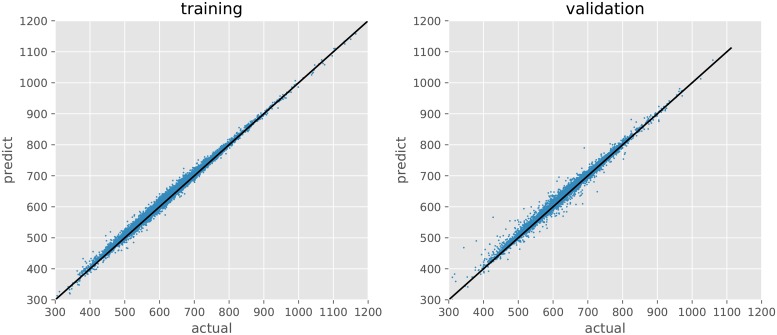
Actual and predicted grain yields in training and validation. MSE between the actual and predicted values is 52.9 and 81.7 in the training and validation processes, respectively.

Fig 3 shows the relation between the actual and predicted grain yields in the training and validation processes. The predictions are considerably close to the actual values in both processes. MSE between the actual and predicted values is 41.5 and 68.2 in the training and validation processes, respectively.

### Model visualization

**Fig 4:**
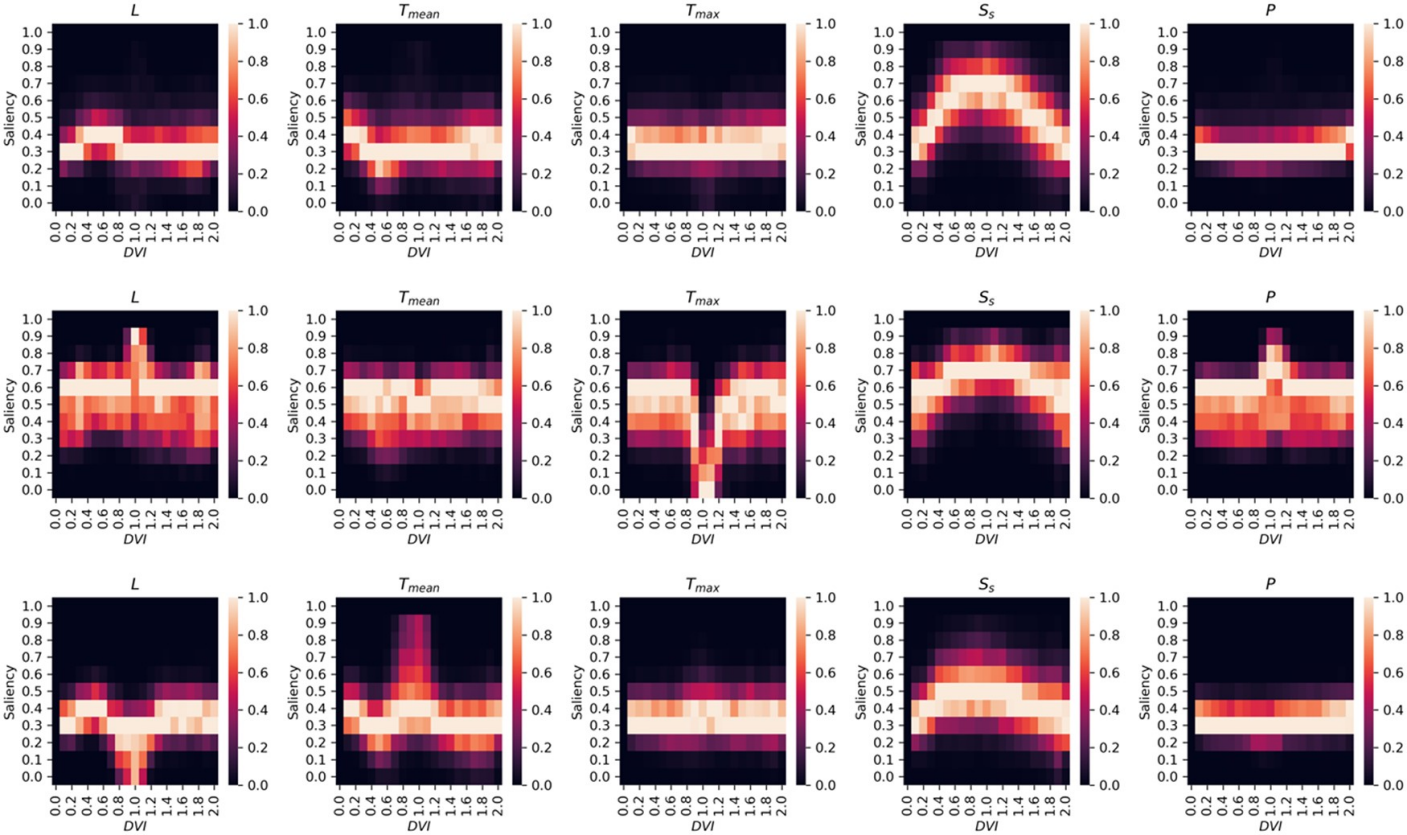
Positive saliencies of different meteorological elements and *DV I*. Saliencies were calculated from 500 datasets that were randomly selected from (top) all datasets, (middle) datasets where $h_{\gamma_{H}}<0.36$, and (bottom) datasets where $h_{\gamma_{L}}<0.35$.

Fig 4 represents the relations between saliency of environmental elements and *DV I*, which represents the growing stage of paddy rice. Figure panels at the top in Fig 4 show the saliencies calculated using all datasets, while those at the middle and bottom are using datasets with spikelet sterility due to high temperature at anthesis and cool summer damage, respectively. The saliencies of environmental elements change along with *DV I*. Moreover, there are some differences in the saliency of each environmental element when different datasets were provided.

## Discussion

Herein, distillation of crop models was conducted to investigate the ability of deep learning to find the plant physiology knowledge behind the distilled crop model from given data. Although most research utilizing machine learning in agriculture lacks sufficient data, this problem was overcome via simulations using crop models. Interestingly, the performance of the model generated by the distillation was analyzed. In addition, the learnings obtained by the model and determination of whether there were any cues related to plant physiological theories behind the distilled crop model were analyzed. CNN, a state-of-the-art method based on deep learning, was used for distillation.

In this study, saliency was calculated using all datasets (case 1) and datasets that faced spikelet sterility due to high temperature at anthesis (case 2) or cool summer damage (case 3). In case 1, the positive saliency of *S*_*s*_ increases rapidly at 0.2 ≤ *DV I* ≤ 1.0. Subsequently, the saliency of *S*_*s*_ decreases again. In fact, in SIMRIW, it is defined that *S*_*s*_ is a function of leaf area index; the leaf area index increases until *DV I* reaches 1.0, after which it starts to decrease. In contrast, the positive saliency of *T*_*mean*_ decreases considerably, which means that negative saliency increases and has some influence on the grain yield at 0.4 ≤ *DV I* ≤ 0.7. At this range of *DV I*, Koshihikari rice growth is affected by *T*_*mean*_ as well as *L*. Although the saliency of *L* does not increase within the range, that of *S*_*s*_, an environmental element similar to *L*, does increase. These results indicate that CNN find a developmental index similar to *DV I*. Moreover, CNN find the important individual meteorological factors affecting the grain yield with the developmental index. Based on these results, CNN is shown to be capable of finding the plant physiology knowledge behind SIMRIW simply by learning climate and plant growth (grain yield) data.

In case 2, the positive saliency of *T*_*max*_ decreases to almost 0 at 0.9 ≤ *DV I* ≤ 1.2. In fact, the percentage of spikelet sterility due to high temperature at anthesis is determined by an accumulated daily maximum temperature in the range 0.96 ≤ *DV I* ≤ 1.2 in SIMRIW. In case 3, the positive saliency of *T*_*mean*_ is found to have slightly increased in the range 0.7 ≤ *DV I* ≤ 1.2. In SIMRIW, cool temperature stress concerning spikelet sterility was measured based on the daily mean temperature in the range 0.75 ≤ *DV I* ≤ 1.2. These results indicate that although CNN has no prior knowledge of spikelet sterility, it realizes that paddy rice becomes sensitive to *T*_*mean*_ or *T*_*max*_ during certain periods. Similar to the results of case 1, CNN successfully find the plant physiology theories behind the crop model simply by learning.

The results demonstrate that machine learning can find plant physiology theories simply by learning climate and plant growth data generated by a crop model without any explicit modeling of the underlying theories. This approach may be helpful for understanding the basic theories behind crop models. For instance, Fig 4 makes it easy to understand the importance of each environmental factor input to SIMRIW in the range of *DV I*. Moreover, the results indicate that machine learning has the potential to discover new theories, even for crops whose plant physiological theory is not revealed yet, simply by learning.

Explaining some saliencies is a challenging task. For instance, the saliency of *P* suddenly increases at 0.9 ≤ *DV I* ≤ 1.2 only when datasets with higher *γ*_*H*_ were provided. Such a theory was not implemented in SIMRIW. However, SIMRIW has multiple empirical parameters that are difficult to understand and such saliencies may be related to these parameters.

In addition, machine learning can accelerate crop growth simulation. The CNN and SIMRIW models required 3.1 ms ± 37 *μ*s and 782 ms ± 2.12 ms, respectively, to produce prediction from 10 datasets. Since the trained model is saved in the Keras format, it can be easily used from Python and JavaScript by employing TensorFlow.js [54] to convert crop models into web applications. Moreover, the proposed approach can be applied to any crop model (even if it is complex) to make the model easier to use and more portable.

Due to ongoing climate change, the agricultural skills and knowledge accumulated over the centuries may not be beneficial in the near future. Thus, data and artificial intelligence methods are needed to improve farming methods. Although machine learning requires big data, which cannot be easily obtained in agriculture due to growing times, this limitation can be overcome using crop models to generate big data on crop growth. In the future, real big data are required to assess the ability of machine learning to discover new plant physiology theories. To this end, it is essential to determine how to effectively collect data on cultivation environments, crop growth, and cultivation management farmers conducted using IoT technologies, which are rapidly developing.

## Supporting information

S1 Fig Schematic showing processes of growth and development and yield formation of rice implemented in SIMRIW [35]. Refer to S1 Table for details of the variables.
(TIF)

S2 Fig Architecture of the CNN model. (EPS)

S3 Fig Time and spatial distributions of training and validation data. Black and red pixels represent the data used for training and validation, respectively.
(TIFF)

S4 Fig Loss in training and validation at different noise levels: (left) training; (right) validation. (EPS)

S1 Table Abbreviations used in this manuscript. (PDF)

S2 Table Minimum loss in training and validation at different noise levels. (PDF)
