# Supplementary figures and images for: Distillation of crop models to learn plant physiology theories using machine learning
Source: PLoS One. 2019 May 29;14(5):e0217075. doi: 10.1371/journal.pone.0217075 (PMC6541271; doi:10.1371/journal.pone.0217075)

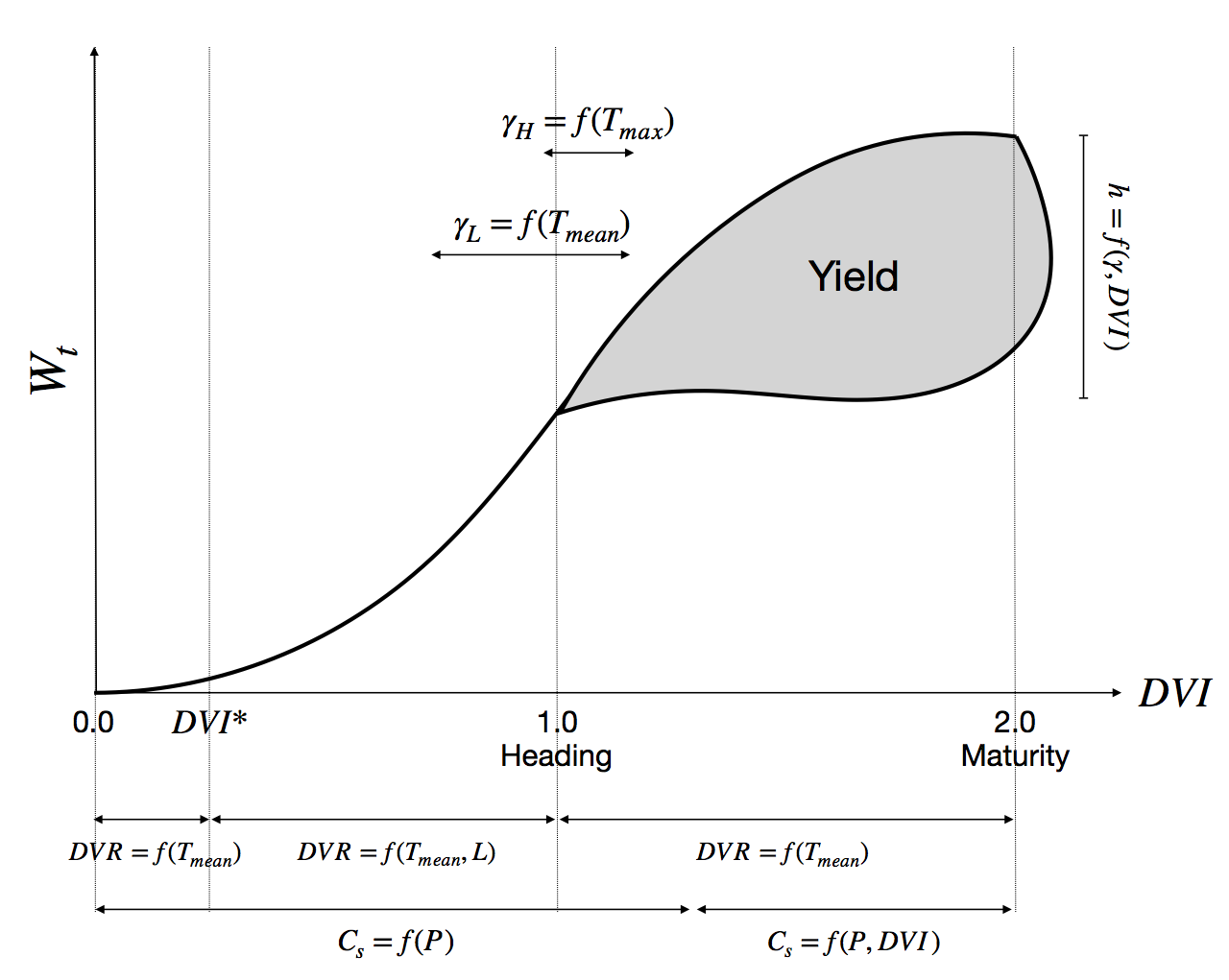

Supplement: S1 Fig — Refer to S1 Table for details of the variables. (TIF) [file pone.0217075.s001.tif]

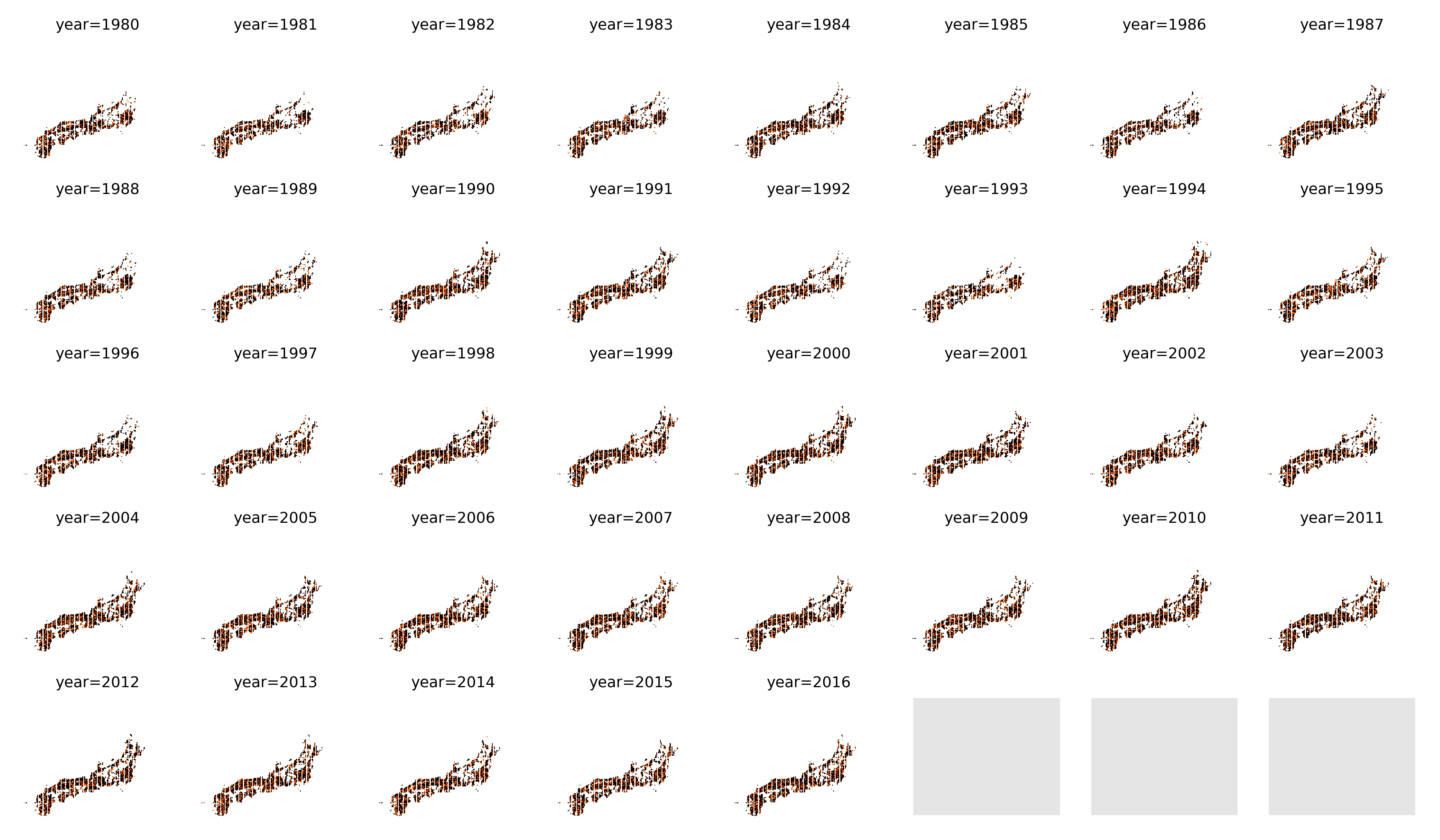

Supplement: S3 Fig — Black and red pixels represent the data used for training and validation, respectively. (TIFF) [file pone.0217075.s003.tiff]
