# Supplementary material for: Distillation of crop models to learn plant physiology theories using machine learning
Source: PLoS One. 2019 May 29;14(5):e0217075. doi: 10.1371/journal.pone.0217075 (PMC6541271; doi:10.1371/journal.pone.0217075)
Supplement: S1 Table — (PDF) [file pone.0217075.s005.pdf]

DVI	Developmental index
DVR	Developmental rate
Y_G	Grain yield
W_t	Total dry matter production
C_s	Conversion efficiency of absorbed short-wave radiation
C_0	C_s at 330 ppm
S_s	Daily total absorbed radiation [MJ/m^2]
h	Harvest index
h_m	Maximum harvest index
h_{γ_H}	Harvest index considering γ_H
h_{γ_L}	Harvest index considering γ_L
T_{mean}	Daily mean temperature [°C]
T_{max}	Daily maximum temperature [°C]
T_H	Average of T_{max} at $0.96 < DVI \leq 1.22$
T_h	Temperature at which DVR is half the maximum rate
L	Day length [h]
L_c	Critical day length
P	CO_2 concentration [ppm]
R_m	Asymptotic limit of relative response to CO_2
G_v	Minimum number of days required for heading
G_r	Minimum number of days required for the grain-filling period
γ	Percentage of spikelet sterility
γ_L	γ due to cool summer damage
γ_H	γ due to high temperature at anthesis
γ_0	Empirical constant
$\mathrm{K_h}$	Empirical constant
$\mathrm{K_q}$	Empirical constant
$\mathrm{K_c}$	Empirical constant
$\mathrm{K_r}$	Empirical constant
$\mathrm{T_{cr}}$	Empirical constant
A	Empirical constant
C	Empirical constant
B	Empirical constant
t	Empirical constant
