# Supplementary material for: Distillation of crop models to learn plant physiology theories using machine learning
Source: PLoS One. 2019 May 29;14(5):e0217075. doi: 10.1371/journal.pone.0217075 (PMC6541271; doi:10.1371/journal.pone.0217075)
Supplement: S2 Table — (PDF) [file pone.0217075.s006.pdf]

Noise level	Training	Validation
$[-0.0, 0.0]$	0.000028	0.000056
$[-0.1, 0.1]$	0.000116	0.000415
$[-.0.01, 0.01]$	0.000027	0.000057
$[-0.001, 0.001]$	0.000030	0.000050
